# Focused Ion Beam Milling Reveals the Role of Crystal Planes in Perovskite Self-Healing

**DOI:** 10.1021/acs.nanolett.6c01531

**Published:** 2026-06-17

**Authors:** Noam Veber, Lotte Kortstee, Roi Gil, Moran Ziv, Betty Shamaev, Saar Shaek, Emma H. Massasa, Shai Levy, Galit Atiya, Juan Maria García Lastra, Ivano E. Castelli, Yehonadav Bekenstein

**Affiliations:** † Department of Materials Science and Engineering, Technion - Israel Institute of Technology, 32000 Haifa, Israel; ‡ The Solid-State Institute, 26747 Technion - Israel Institute of Technology, 32000 Haifa, Israel; § The Resnick Sustainability Center for Catalysis Institution, 26747 Technion - Israel Institute of Technology, 3200008 Haifa, Israel; ∥ Department of Energy Conversion and Storage (DTU Energy), 5205 Technical University of Denmark, Agnes Nielsens Vej 301, DK-2800 Kongens Lyngby, Denmark; ⊥ Physics Department, 153960 Technion - Israel Institute of Technology, 32000 Haifa, Israel

**Keywords:** self-healing, halide perovskites, surface energy, vapor deposition, focused ion beam

## Abstract

Self-healing is rarely observed in semiconductors, where structural distortions typically result in an irreversible performance loss. Halide perovskites defy this paradigm, exhibiting spontaneous recovery of optoelectronic properties even at room temperature, yet the underlying mechanisms remain poorly understood. Here, we subject CsPbBr_3_ single crystals to facet-oriented focused ion beam (FIB) milling to induce localized mechanical damage and directly track the subsequent healing dynamics. By selectively exposing different crystallographic orientations, we correlate structural reconstruction with photoluminescence recovery. Milling aligned with low-index surfaces enables complete recovery, often with enhanced emission compared to that of the pristine surface, whereas milling across facets, along effectively higher-index crystal planes, leads to permanent emission quenching. The differences arise due to the facet-dependent stabilization and higher formation energies of Br interstitials for higher-index surfaces, a hypothesis that is supported by DFT modeling. Our work establishes facet-oriented FIB milling as a versatile approach for systematically probing self-healing processes in functional materials.

## Graphical abstract

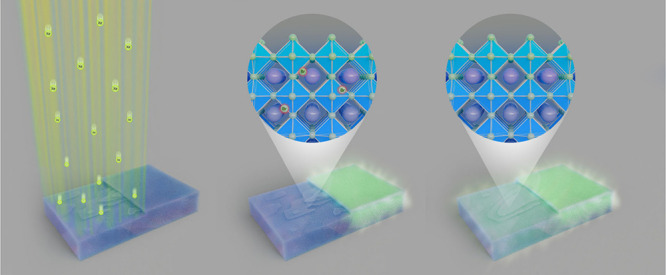

Unlike traditional bulk semiconductors, which are structurally rigid and exhibit a limited capacity for self-healing,^1^ halide perovskites display an unusual ability to undergo spontaneous annealing (self-healing) even at room temperature.^2–5^ This behavior originates from their dynamic crystal lattice,^6–8^ low melting temperatures,^9^ enhanced diffusion,^10,11^ accommodation of strain,^12^ and spontaneous structural rearrangement processes.^13–15^ These characteristics underpin the exceptional electro-optical properties of halide perovskites, including tunable band gaps and low-cost fabrication,^9,16–18^ making them promising materials for next-generation photovoltaics and light-emitting diodes.^19–21^ However, despite these advantages, achieving long-term structural and optical stability remains a central challenge.^22–25^

Halide perovskites have been shown to exhibit self-healing behavior in their structures and optoelectronic properties.^26–29^ This includes a wide variety of damage sources such as photodamage,^30–33^ electrical bias or ion migration-induced damage,^34–36^ moisture-related effects,^37^ and mechanical damage.^3,38^ Typically, self-healing is studied by intentionally inducing damage in the crystal and monitoring its subsequent recovery. Photodamage using high-intensity excitation is the most widely used method for this purpose, offering a flexible means to distinguish between surface and bulk effects while enabling real-time observation of healing dynamics.^2,27,30^ Other techniques include high-energy radiation damage such as X-ray,^39,40^ proton,^41,42^ and electron irradiation.^8,43^

Self-healing from mechanical damage has previously been demonstrated through the application of localized force using an atomic force microscopy (AFM) tip^3,38^ as well as through the introduction of cracks into the material.^28,29^ In contrast to many other degradation pathways discussed above, mechanically induced damage is typically confined to the surface or interfacial regions of the material, leading to distinct recovery dynamics. For instance, in CsPbBr_3_, increased humidity was shown to accelerate the healing process after mechanical damage, whereas MAPbBr_3_ exhibited little to no humidity dependence under similar conditions.^3^ These observations emphasize the important role of surface chemistry and interfacial energetics in governing the self-healing processes.

Focused ion beam (FIB) milling offers another avenue for inducing localized mechanical damage with the additional capability of patterning complex nanostructures for photonic applications.^44,45^ However, to the best of our knowledge, FIB has not been applied to directly study self-healing in halide perovskites. The advantages of using FIB milling include the ability to induce structural perturbations with atomic-scale precision and deliberately expose high-index or low-index crystal facets for systematic investigation of surface-dependent healing dynamics. While the milling process inherently removes material through sputtering, the resulting damage and subsequent self-healing occur within the crystal lattice surrounding the milled region. This level of control enables direct access to surface orientations and geometries that are difficult to isolate using conventional mechanical damage methods, such as indentation.

Most research on self-healing has focused on polycrystalline films, which are highly relevant for device applications but limit the ability to isolate the healing capability of different facets. Alternatively, single crystals offer a well-defined structure for probing the fundamental mechanisms of the crystal self-healing process and optical recovery. The surface chemistry of halide perovskites plays a pivotal role in their optoelectronic behavior.^46–49^ Different crystal facets possess unique chemical and physical characteristics that can significantly influence the dynamics of structural rearrangement and healing.^50–53^ In a previous study by us on single crystals at the nanoscale, the surface energy and facet passivation have played a pivotal role in self-healing.^8^ Therefore, a deeper understanding linking facet-specific surface termination to the self-healing process and outcomes is of great interest for enhancing the long-term stability of these materials.

In this work, we use FIB milling to selectively remove material and induce localized structural distortions in a controlled manner. The samples we use are CsPbBr_3_ single crystals grown via vapor deposition, and we study the recovery by measuring their PL response over time and monitoring their surface reconstruction using AFM. By comparing different FIB milling geometries, we find that self-healing occurs only when milling is aligned with the crystal facet rather than cutting across it and exposing higher-index planes. The observed difference arises from different surface energies between the two cases, leading to the stabilization of Br interstitials on higher-index planes, preventing their optical recovery. These claims are further supported by density functional theory (DFT) calculations, stressing the stabilization of the Br interstitial defect as a contributor to this process. Together, these findings highlight the critical role of facet orientation in the self-healing process, offering new strategies for improving the long-term stability of halide perovskite-based devices through facet engineering.

CsPbBr_3_ crystals were grown using vapor deposition (see the Supporting Information for further details), and the resulting crystals are a few micrometers in size and predominantly rectangular in shape. They are characterized by bright emission around 530 nm (Figure S1). In order to study the self-healing capability of CsPbBr_3_ microcrystals, we applied FIB milling. In this way, we are able to remove material with a predetermined pattern from the crystal face (see Figure 1a–d). We then systematically investigated how the properties of the removed area affect the self-healing process. We take extra caution not to alter the crystal composition by using a xenon ion beam and work at low currents to reduce heating effects and maintain good control over the sputtering rate. This is further verified by using EDS (for more information see Methods in the Supporting Information and Figure S2). The resolution of FIB milling methodology together with the highly defined samples enables us to expose selected crystal facets. For example, for (100) and (110), these are known to possess different terminations and therefore different surface energies.^54^ In return, they are expected to affect the rate and effectiveness of the healing process.

**Figure 1:**
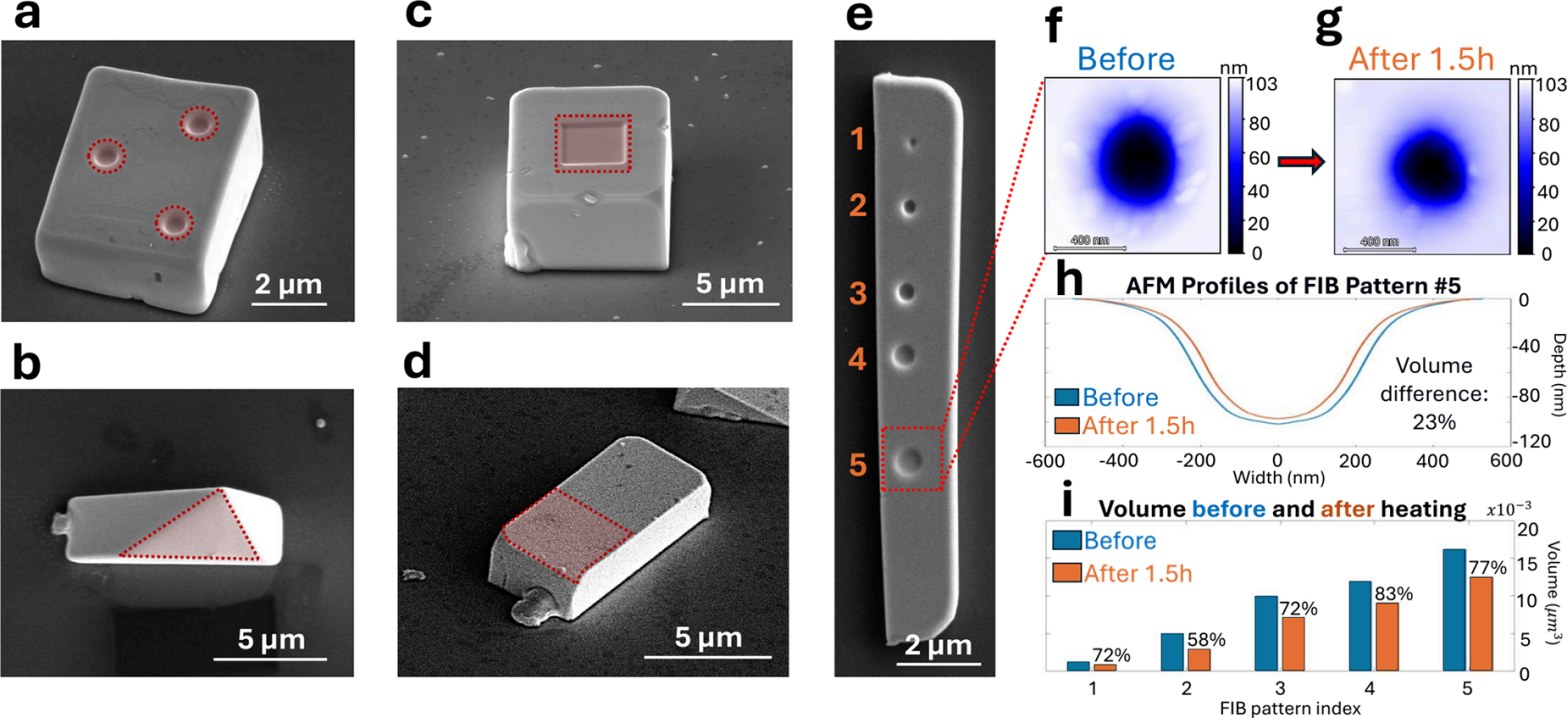
Examples of different FIB patterns on single microcrystals of CsPbBr_3_ exposing different facets and geometries: (a) circular FIB patterns, (b) rectangular FIB pattern, (c) milling at an angle to expose higher-index facets, and (d) FIB milling on one half of the crystal for easy comparison of the damaged part and the intact crystal. (e) Circular FIB patterns on a single microrod of different diameters, marked by indices 1–5 from the smallest to the largest. (f and g) AFM topography of circular pattern 5 before and after annealing at 100 °C, demonstrating a clear reduction in size and surface rearrangement. (h) AFM topography profiles of pattern 5 before and after annealing. From these measurements, a significant reduction in volume of 23% is calculated after annealing. (i) Comparison of the volume before and after annealing for FIB patterns 1–5, showing clear reduction in volume in all cases.

Our first aim is to test the limits of surface reconstruction after FIB damage. For this purpose, we etched circular patterns of different volumes along a single microcrystal (Figure 1e). The diameters of the patterns range from 100 to 500 nm, and their depths from 50 to 200 nm. After annealing for 1.5 h at 100 °C under ambient conditions, the volume of all etched patterns shrunk by around 25%, indicating that surface reconstruction of large, damaged areas is possible (further details in Figure S3).

Having established that substantial surface reconstruction occurs following FIB structural distortions, we next want to explore the structure function relations between the etched facets and resulting emission properties of the crystals. For ease of comparison, we first focus on patterns that expose only half of the crystal to FIB damage, as shown in Figure 1d and illustrated in Figure 2a. In this line of experiments, we remove material from the crystal surface via sputtering, thereby damaging half of the surface while leaving the other half intact. The dominant defects expected under these conditions arise from atomic displacements, primarily vacancies and interstitials. In CsPbBr_3_, the bromide is the weakest link, since it is both lighter and more abundant than Cs and Pb.^55^ Furthermore, the formation energy of a Br vacancy and interstitial is lower than those of Cs and Pb.^56^ We therefore expect that Br interstitials, known for having a detrimental effect on the emission,^57–59^ will dictate the dynamics of self-healing (see the illustration in panels b and c of Figure 2). Immediately after FIB milling, we measure the emission from the crystals, comparing the intensity between the etched half and the unperturbed one. It is apparent that the etched side of the crystal suffers a significant reduction in the intensity of the emission, while the undamaged half continues to emit brightly (see Figure 2d). In this way, we can use the unaffected half of the crystal as the internal standard to quantify the emission recovery after a self-healing process (see Methods in the Supporting Information). We observe that the crystal regains its emission on a time scale of hours to a few days. Figure 2e shows the same crystal from Figure 2d after 24 h, demonstrating an almost complete recovery of the optical properties (more examples and additional information in Figures S4 and S5).

**Figure 2:**
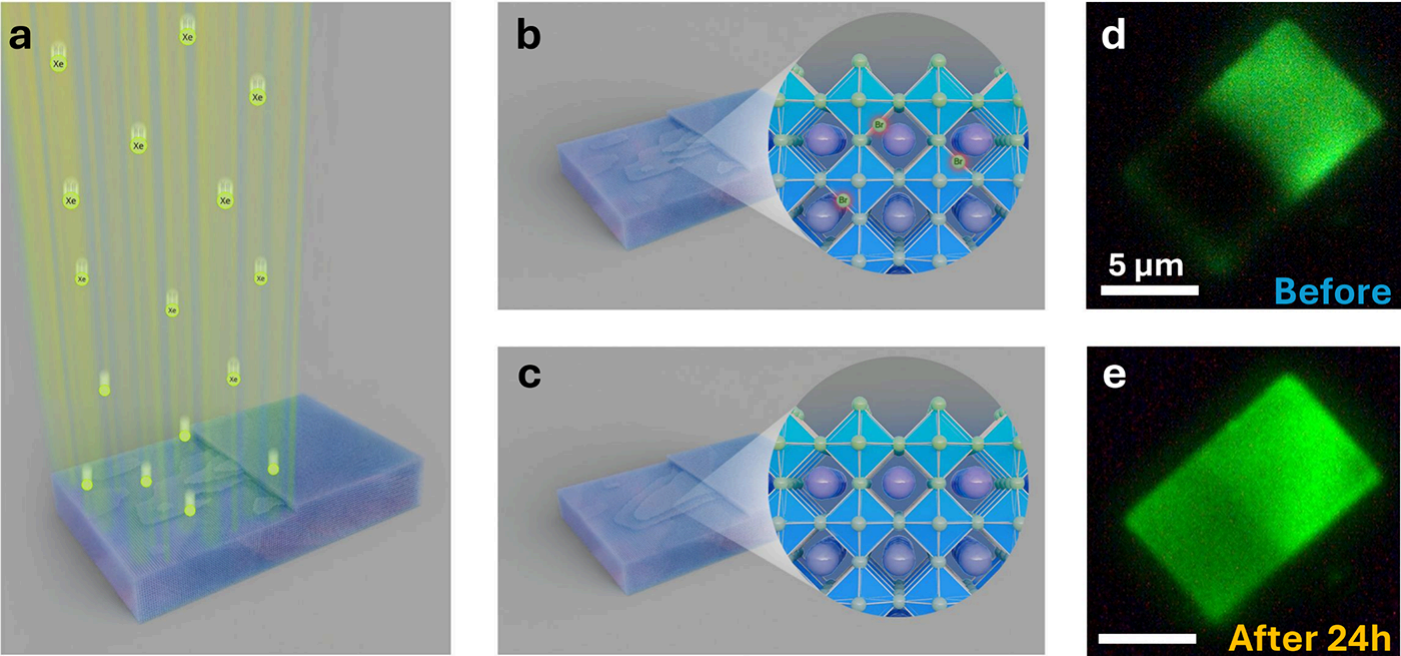
(a) Illustration of FIB-induced damage to one half of the crystal, leaving the other half intact that serves as a reference. (b) Illustration of the damage to the crystal’s surface, showing the introduction of many Br interstitials (inset), leading to reduced emission. (c) Illustration of the damaged surface after recovery, showing a more ordered surface and the elimination of the Br interstitials (inset). (d) Emission of a particle after half of the crystal was damaged by FIB, showing reduced emission on the etched side, and bright emission on the unaffected side. (e) Emission of the same particle from panel d after 24 h, showing recovery of the emission.

To understand the recovery and correlate structure–function relations in this process, we analyzed crystals in which only one half was exposed to FIB damage while the other half remained intact. The most apparent difference between the etched and intact parts is the emission intensity. The emission from the FIB-etched half is entirely quenched, while the unexposed half retains its original photoluminescence (see Figure S6). Using AFM, we characterized the surface morphology post-FIB treatment and compared it with the adjacent unperturbed region (intact). Figure 3a shows the AFM amplitude micrograph of such a crystal, revealing a clear boundary and a difference between the etched and intact regions. Interestingly, the height difference between the two regions (Figure 3b) is only ∼3 nm, significantly smaller than the total crystal height of 600 nm (see Figure S7), and corresponds to removal of only five or six unit cells. We thus conclude that the emission quenching in the etched area is a purely surface-based effect, since the FIB has removed only a thin layer from the surface. Such a pronounced effect can be attributed to the electron diffusion length in halide perovskites, which is approximately 1 μm,^60,61^ much larger than the crystal thickness, implying that surface damage can influence the emission response from a large volume. A more detailed inspection of the surface topography (Figure 3c) reveals two notable features. First, we observe step-like terraces (outlined with dotted blue lines), present on the etched and intact regions. After FIB milling, the terrace area appears to be significantly smaller. The step height, measured to be ∼0.6 nm (Figure 3d), corresponds closely to the lattice parameter of CsPbBr_3_, indicating that FIB milling in CsPbBr_3_ can achieve removal precision at the unit cell level. Second, we observe the existence of small particulates (1–2 nm, highlighted with a blue circle) only in the unexposed region. Based on previous studies,^53^ we assign these features to lead precipitates that appear on these exposed surfaces with time. The observation of these features only on the unperturbed surfaces suggests that FIB milling can selectively remove these precipitates from the perovskite surfaces, rendering these surfaces closer to a perfect perovskite surface. Indeed, in several cases (Figure 3e–g), we find that the damaged area recovers to stronger emission values compared to the same region prior to etching. This phenomenon of enhanced emission following recovery has previously been reported in several self-healing studies of halide perovskites,^2,26,27,30^ albeit with different damage sources. We therefore sought to discover the mechanism behind this enhanced emission in our case.

**Figure 3:**
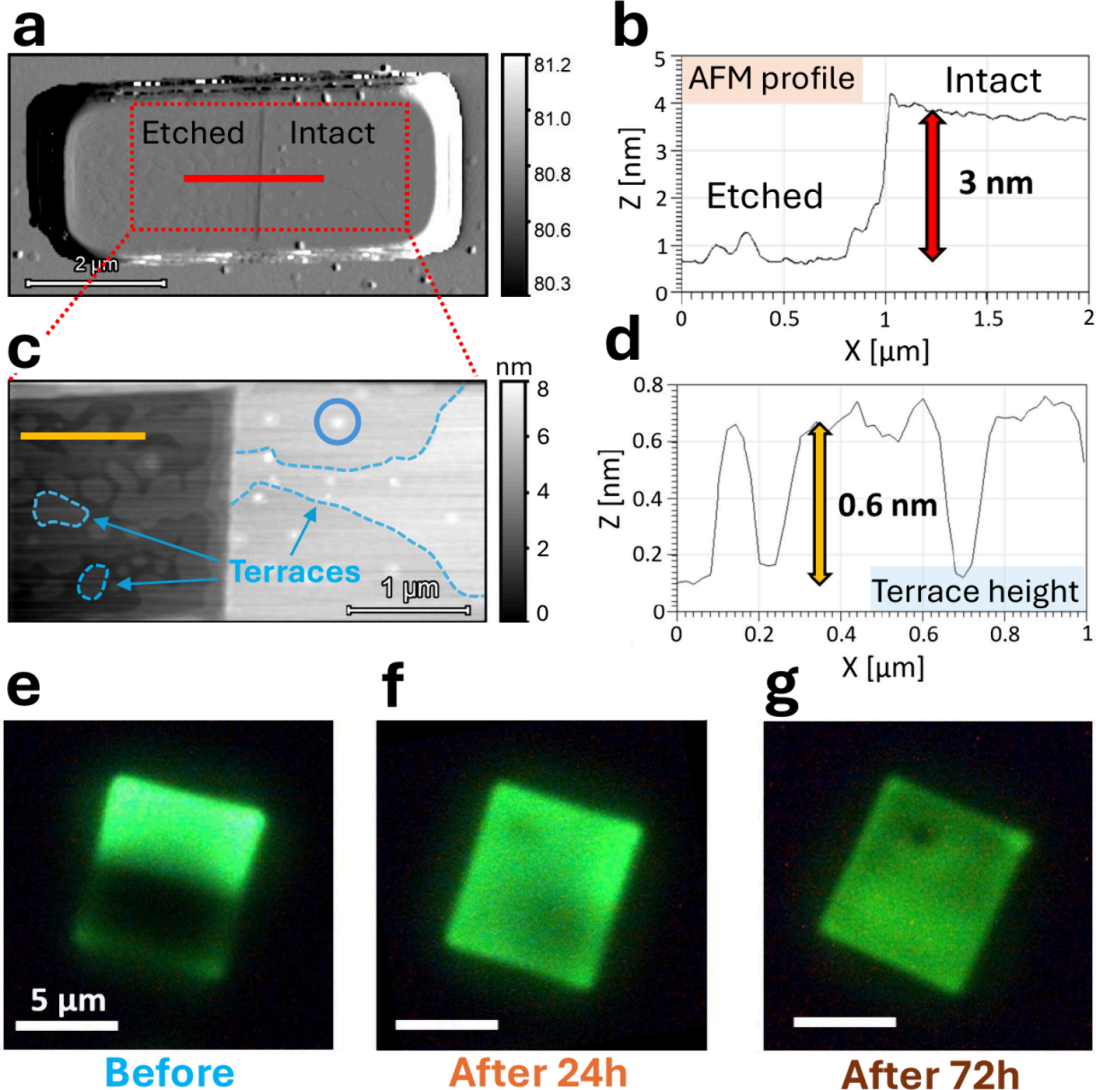
(a) AFM amplitude micrograph of the CsPbBr_3_ microcrystal after FIB damage, showing the FIB-milled (etched) and intact regions. (b) Line profile of the topography as indicated by the red line in panel a, showing a 3 nm difference between the etched and intact regions. (c) AFM topography of the crystal’s surface, showing narrow terraces on the damaged half and wide terraces on the side that remained intact (highlighted by a dotted blue line). A blue circle on the unaffected half highlights defects that are eliminated after FIB. (d) Line profile along a few terraces showing that their height corresponds to the unit cell of CsPbBr_3_. (e) Emission of a particle right after FIB (before healing) and its emission after (f) 24 and (g) 72 h, showing recovery of the damaged facet that eventually emits more brightly than the facet that remained intact.

Since the FIB damage is confined primarily to the crystal surface, we can use AFM to monitor surface evolution during the healing process and relate it to changes in the optical properties. Panels a and b of Figure 4 show the surface topography of a crystal immediately after FIB milling and again after 3 days. Initially, numerous small terraces are visible on the etched region (highlighted in yellow), while the intact region displays larger, well-defined terraces (highlighted in red). After 3 days, the number of terraces on the etched side noticeably decreases, and many of the remaining ones appear with a smaller area. In contrast, the terrace structure on the untreated side remains largely unchanged over the same period. To quantify this surface evolution, we traced the perimeters of the terraces (step length) on both sides of the crystal and compared their total lengths before and after the healing period. As shown in panels c and d of Figure 4, the cumulative step length on the etched side is reduced by more than 30%, indicating significant surface reconstruction (for an additional example, see Figure S8). Meanwhile, the intact region showed no appreciable change. This substantial reduction in step length suggests that the damaged surface undergoes a spontaneous reconstruction process, moving toward a more thermodynamically stable configuration relative to the original undamaged crystal, which also eliminates defect electronic states that were inevitably embedded in the parent crystal. Correspondingly, photoluminescence (PL) measurements (Figure 4e,f) reveal not only complete recovery but also enhanced emission from the etched region after 3 days.

**Figure 4:**
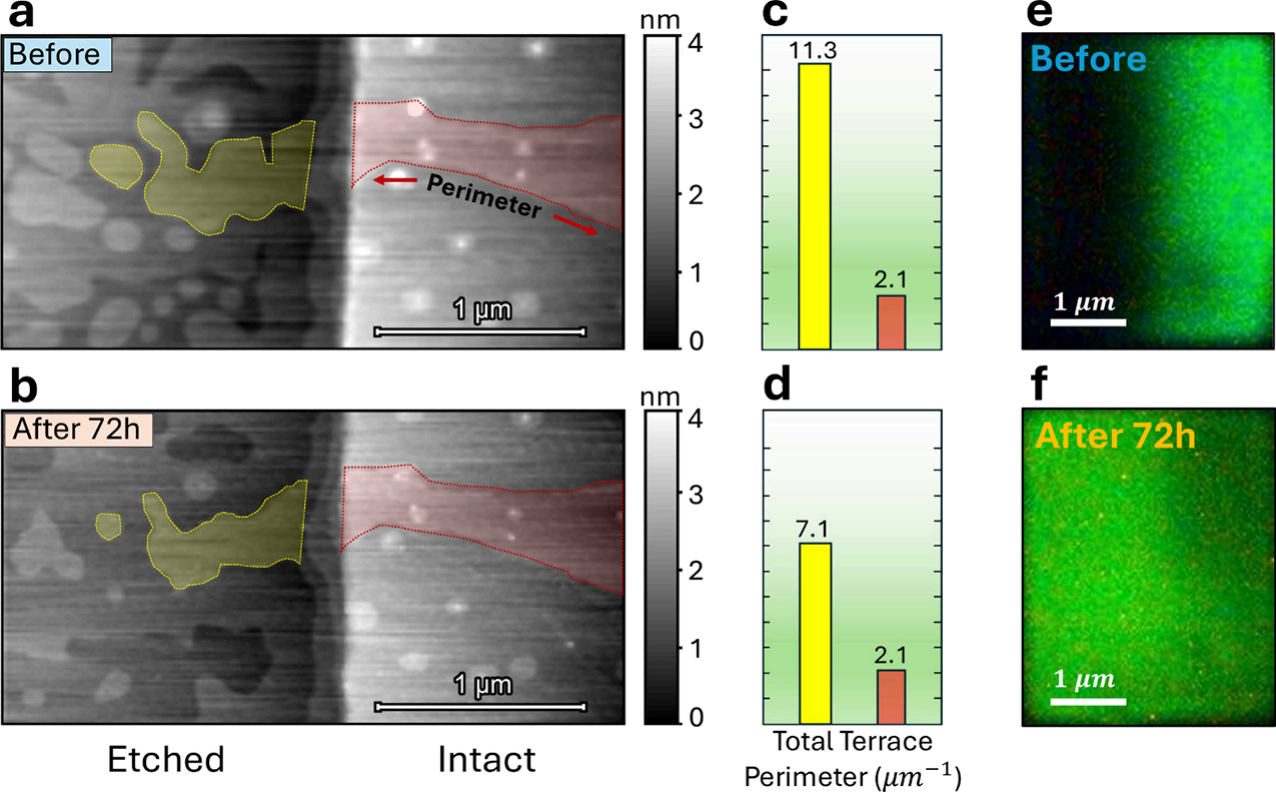
(a) AFM topography of a crystal’s surface right after FIB milling (before) and after 72 h in panel b, showing a comparison between the two sides of the crystal. The terraces on the unexposed side are colored red and yellow on the etched side, showing substantial surface reconstruction. The cumulative step lengths (perimeter) of the two sides are compared (c) right after FIB and (d) after 72 h, showing a clear reduction for the etched half, indicating crystal reconstruction. The perimeter is normalized, displaying the total terrace perimeter per unit area. (e) PL right after FIB, showing quenching of the emission across the etched part. (f) PL after 72 h, showing recovery of the emission. The etched side emits brighter after the healing process.

To highlight the role of surface reconstruction and the crystal’s intrinsic ability for self-healing, we compare the emission behavior of two crystals subjected to different FIB patterns. Both patterns are circular in shape; however, in the first case (Figure 5a), the crystal fails to recover its emission (dark case), while in the second case (Figure 5b), the emission fully recovers (bright case). To investigate the origin of this difference, we performed AFM measurements focusing on the profiles of the circularly milled regions (Figure 5c,d). The corresponding height profiles are shown in panels e and f of Figure 5. These profiles reveal a clear distinction between the dark and bright cases. In the dark case, FIB milling was misaligned with the facet of the perovskite and was executed at an oblique angle to the ⟨100⟩ primary crystal facets, exposing high-index crystallographic planes in the walls of the milled hole. However, for the bright case, FIB milling was typically aligned with the ⟨100⟩ facet (i.e., along crystal facets). This resulted in mainly exposing lower-index facets in the milled hole. These observations, together with the observation of surface reconstruction, suggest that milling along crystal facets facilitates structural rearrangement toward a more thermodynamically stable configuration, enabling full optical recovery. Conversely, milling across facets leads to a less stable surface structure that hinders recovery of the optical properties.

**Figure 5:**
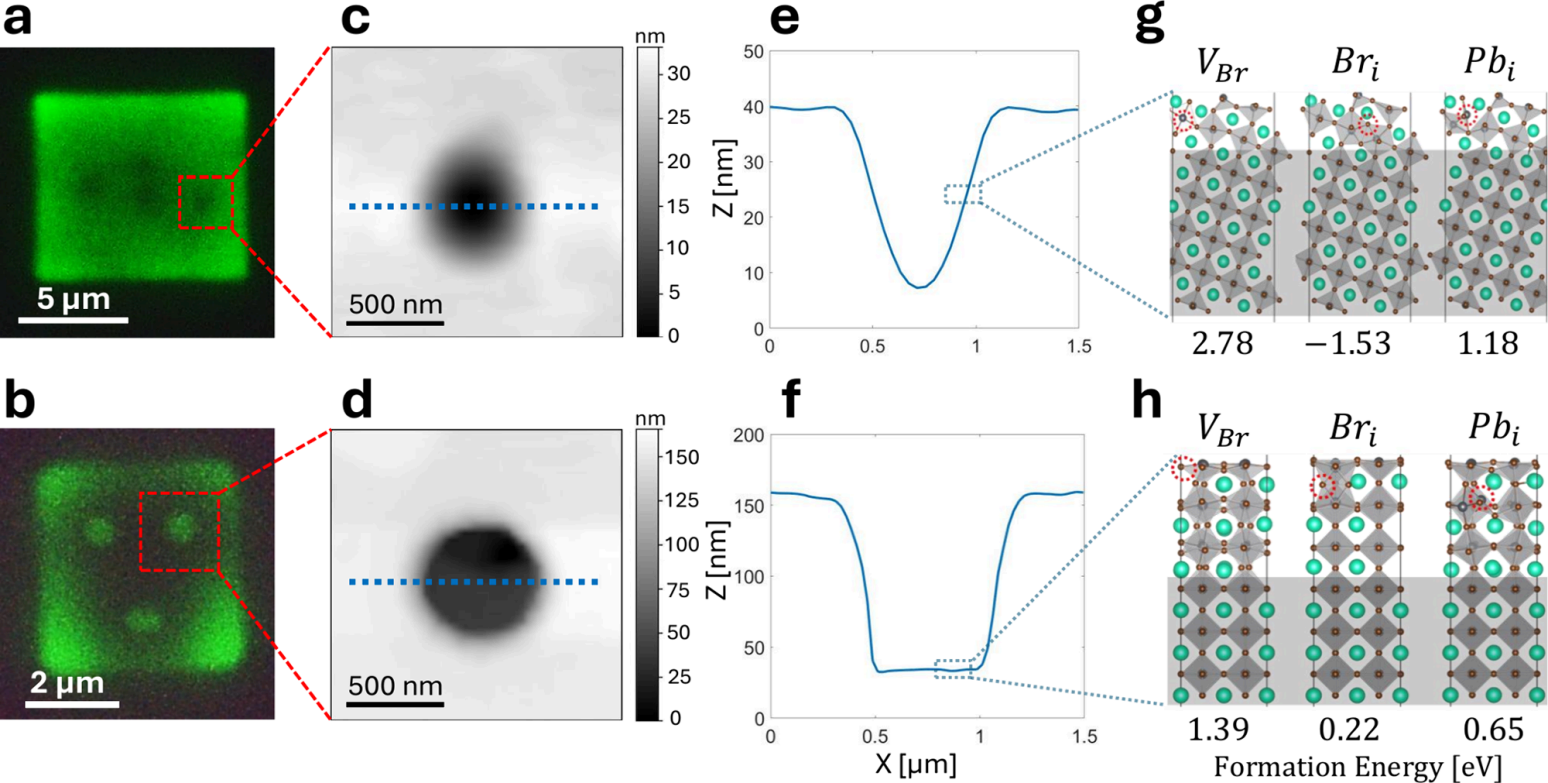
PL of two crystals with circular FIB patterns after a healing period. In panel a, the crystal appears to be unable to self-heal even after many days (dark case), while in panel b, complete recovery is observed after 24 h (bright case). (c and d) AFM topography of the circular patterns presented in panels a and b, respectively. (e) Line profile of the topography along the FIB pattern in panel a, where the surface of the pattern is not aligned with the original surface of the crystal (cross facet), preventing self-healing. (f) Line profile along the FIB pattern in panel b, where the surface of the FIB pattern is aligned with the original surface of the crystal (along facet), allowing self-healing. (g and h) DFT calculations for the formation energies of defects where the cut is along the (310) facet and across the (100) facet, respectively. The calculations show that the Br interstitial is more likely to form, and its stability is enhanced when the cut is across facet a, preventing self-healing.

To support this hypothesis with quantifiable thermodynamic values, we have performed DFT calculations using the VASP code.^62^ We specifically focused on surface and formation energies for the planes observed for the bright and dark healing cases (computational methods explained in the Supporting Information). We modeled the surfaces approximated by Miller indices (310) for the case shown in Figure 5e (i.e., across crystal facets) and (100) for the well in Figure 5f (i.e., along crystal facets). The DFT results reveal substantial differences in surface energetics between these misaligned facets. The (100) surface (formation energy of 0.86 meV/Å^2^) is energetically favored over the (310) surface (formation energy of 15.33 meV/Å^2^), as detailed further in the Supporting Information (Figure S9). We next evaluated the formation energies of V_Br_, Br_i_, and Pb_i_ defects on these surfaces (Figure 5g,h). These defects were selected because of their expected high concentration in the bulk and their pronounced influence on nonradiative recombination.^56,57,63^ A striking difference in defect behavior is observed for the bromide interstitial, which is strongly stabilized on the (310) surface, with a formation energy of −1.53 eV, compared to 0.22 eV on the (100) surface. With the knowledge that the bromide interstitial can have a significant effect on the emission,^56,57^ the extreme stabilization of Br_i_ as a nonradiative recombination center can provide an explanation for optical recovery in our experiments for cross-facet FIB milling (Figure 5a). On the other hand, Br_i_ defects are less stable on the 100 facet and can thermodynamically be easier to resolve in a self-healing process observed on the 100 facet.

These findings may also help rationalize broader observations regarding interface- and environment-dependent stability in halide perovskites.^64^ Previous studies have shown that local environments,^3^ surface passivation,^8^ and interfacial chemistry^65,66^ strongly influence degradation and recovery dynamics in these materials. In this context, the facet-dependent healing behavior observed here suggests that the enhanced stability commonly reported for low-dimensional and 2D perovskites^67,68^ may arise from not only ligand passivation but also preferential exposure of energetically favorable crystal facets. Since 2D perovskites typically grow along specific low-energy orientations, their improved resistance to degradation may additionally benefit from more efficient self-healing pathways associated with these surfaces.

In conclusion, self-healing in semiconductors is possible, and for halide perovskites, one can even make better crystals using this spontaneous process. By selectively removing specific regions of the crystal and monitoring their optical and structural evolution over time, we demonstrated that halide perovskites can spontaneously recover their emission even when half of the crystal surface is damaged. The most important finding relates the directionality of FIB milling relative to the crystal’s surface facets to healing outcomes. When milling is aligned with the natural crystal facets, the degraded area undergoes complete recovery, often with enhanced emission compared to the original unaffected surface. In contrast, milling across facets leads to misaligned surfaces that are unable to recover, resulting in permanent emission quenching. We show these differences are rooted in thermodynamic stabilization of Br interstitials that are limiting the self-healing process for the higher-index planes. Since facet-dependent surface energetics and ionic defect dynamics are general characteristics of halide perovskites, the mechanism proposed here is likely relevant beyond CsPbBr_3_. These results highlight the importance of surface orientation in guiding the self-healing pathways of halide perovskites and provide a new pathway for enhancing the long-term stability of perovskite-based optoelectronic devices.

## Supplementary Material



## Data Availability

All data are available in the main text or the Supporting Information.

## Notes

Materials and methods, basic characterization, EDS, cathodoluminescence, optical measurements, AFM results, and DFT calculations (PDF)
