# Supplementary material for: Focused Ion Beam Milling Reveals the Role of Crystal Planes in Perovskite Self-Healing
Source: Nano Lett. 2026 Jun 17;26(25):8249–56. doi: 10.1021/acs.nanolett.6c01531 (PMC13329983; doi:10.1021/acs.nanolett.6c01531)
Supplement: Supplementary file 1 [file nl6c01531_si_001.pdf]

Supporting Information for:

Focused Ion Beam Milling Reveals the Role of Crystal Planes in Perovskite Self-Healing

Noam Veber[1,2], Lotte Kortstee[3], Roi Gil[4], Moran Ziv[4], Betty Shamaev[1,2], Saar Shaek[1,2], Emma H. Massasa[1,2], Shai Levy[1,2], Galit Atiya[1], Juan Maria García Lastra[3], Ivano E. Castelli[3], Yehonadav Bekenstein*[1,2]

Affiliations:

[1]Department of Materials Science and Engineering, Technion – Israel Institute of Technology, 32000 Haifa, Israel.

[2]The Solid-State Institute, Technion – Israel Institute of Technology, 32000 Haifa, Israel.

[3]Department of Energy Conversion and Storage (DTU Energy), Technical University of Denmark, Agnes Nielsens Vej 301, DK-2800 Kongens Lyngby, Denmark

[4]Physics Department, Technion − Israel Institute of Technology, 32000 Haifa, Israel

*Corresponding author: Email: bekenstein@technion.ac.il

1. Methods

Materials:

Lead Bromide (99.998%, Alfa Aesar), Cesium Bromide (99.999%, Sigma Aldrich). All chemicals were used as purchased with no further purifications.

Vapor deposition of $CsPbBr_3$ crystals:

The vapor growth process was conducted in a 3-zone tube furnace (Zhengzhou Protech Technology, PT-1200T CVD TubeFurnace) with a 32mm diameter quartz tube, where the precursors, lead bromide and cesium bromide, were taken at a 1:1 ratio, mixed, and placed in the first zone. After cleaning with acetone, ethanol, and isopropanol, the silicon wafers were placed in the second zone, 10cm downstream from the precursors. The tube was degassed three times with nitrogen gas. Then, the pressure and flow were kept at 300 mbar and 400 sccm throughout the entire process. The first heating zone was set to 600°C with a heating rate of 15°C/min, and the second and third zones were set to 385°C. Then, the temperatures were kept for 10 minutes. The furnace was then cooled naturally to room temperature under nitrogen flow, and the cooling rate was 1.5°C/min (from 400°C to room temperature in 4 hours).

Optical measurements – reflection, Photoluminescence (PL):

The optical measurements (UV, reflection, and PL) were done using an Edinburgh Instruments FLS1000 photoluminescence spectrometer coupled to Nikon Eclipse UPRIGHT Ni-U architecture light microscope. The samples were irradiated using a xenon lamp (Xe900), and the measurements were taken in reflection mode.

To monitor the self-healing process, PL measurements were performed immediately after FIB milling in order to capture the initial state of the particles under ambient conditions. Between successive measurements, the samples were stored inside a desiccator, providing a low-humidity, controlled environment that minimizes external environmental influences.

Scanning Electron Microscopy (SEM):

SEM characterization was done using Zeiss Ultra-Plus FEG-SEM for the HRSEM measurements. For composition analysis, samples were placed at a 4mm working distance and measured with acceleration voltage between 2 to 4KV. Energy Dispersive X-ray Spectroscopy (EDS) measurements were taken with an Oxford SDD EDS detector with an energy resolution of 127eV at 10KV and a 7mm working distance.

Plasma Focused Ion Beam (PFIB) Milling:

FIB milling was performed using a Thermo Fisher Helios 5 Plasma Focused Ion Beam (PFIB CXe). The process was carried out at an accelerating voltage of 30 kV, with the stage tilted to 52° to ensure the ion beam was perpendicular to the sample surface. Rectangle/circle patterns were generated using beam currents ranging from 3 to 10 pA, with exposure times varying between 5 and 60 seconds, depending on the specific pattern geometry.

The low beam currents are used in order to maintain good control over the sputtering rate, and to reduce heating effects.

Ion penetrations depth: based on previous works,[1,2] we estimate the penetration depth of the xenon ions at around 10-20 nm. This value is much smaller than the crystals' height, indicating that most of the damage is concentrated close to the surface.

Atomic Force Microscopy (AFM):

Measurements were taken using a Neaspec system in tapping mode, using PPP-EFM tip from NANOSENSORS. Tip radius: <25nm, resonance frequency – 75kHz with PtIr5 coating.

2. Basic Characterization

The resulting particles from our synthesis are of various shapes and emit brightly at around 530 nm as can be seen in Figure S1.

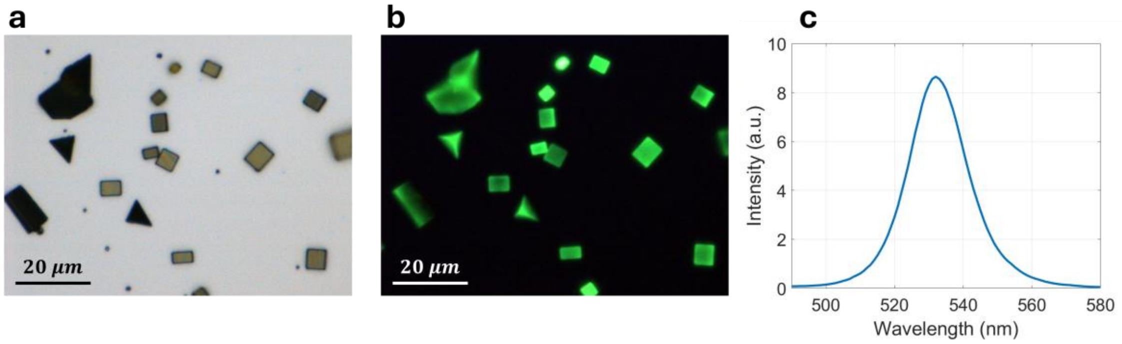

Figure S1: (a) White light optical image of $CsPbBr_3$ particles. (b) The emission of the particles under excitation of 405 nm wavelength. (c) The PL spectra of the particles show an emission peak at 533nm.

3. Compositional Analysis

The composition of the particles after FIB milling was measured using EDS to confirm the material remained the same after the FIB. The results are shown in Figure S2.

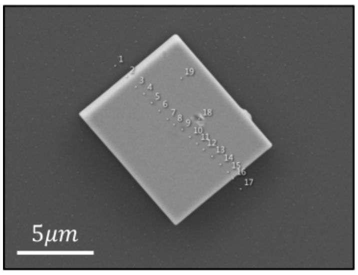

Unaffected surface		FIB pattern		Substrate (Si)	
Element	Atomic %	Element	Atomic %	Element	Atomic %
Cs	4.27	Cs	4.37	Cs	0
Pb	4.28	Pb	4.31	Pb	0.11
Br	11.2	Br	11.3	Br	0.28

Figure S2: SEM EDS composition analysis at the FIB pattern and away from it on the pristine perovskite surface. The measurement shows the expected ratio between the elements, with additional reference measurement on top of the silicon substrate which does not show the perovskite composition.

4. FIB Damage

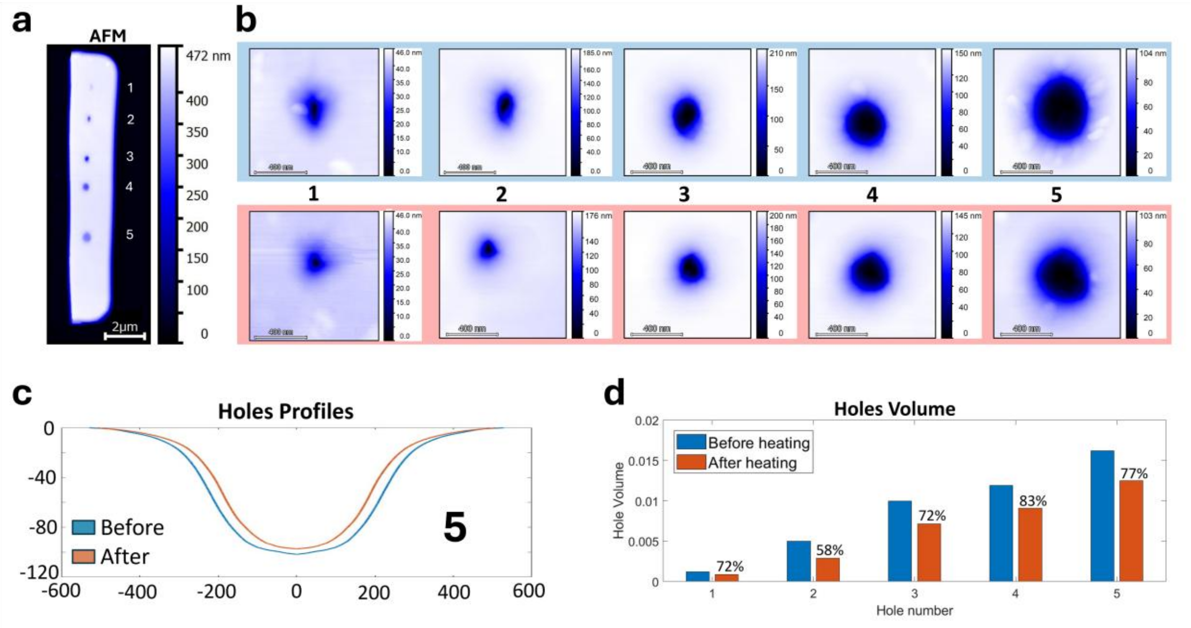

Figure S3 presents FIB damage before and after annealing at 100℃.

Figure S3: (a) AFM topography of a $CsPbBr_3$ microrod with five 'holes' of different sizes. (b) Close up on the holes before heat treatment for 1.5 hours at 100℃ in blue and after heat treatment in red. (c) The profile of hole '5' before and after heat treatment. (d) Comparison of the holes volume before and after heat treatment, showing a reduction of 17-42% in all of them.

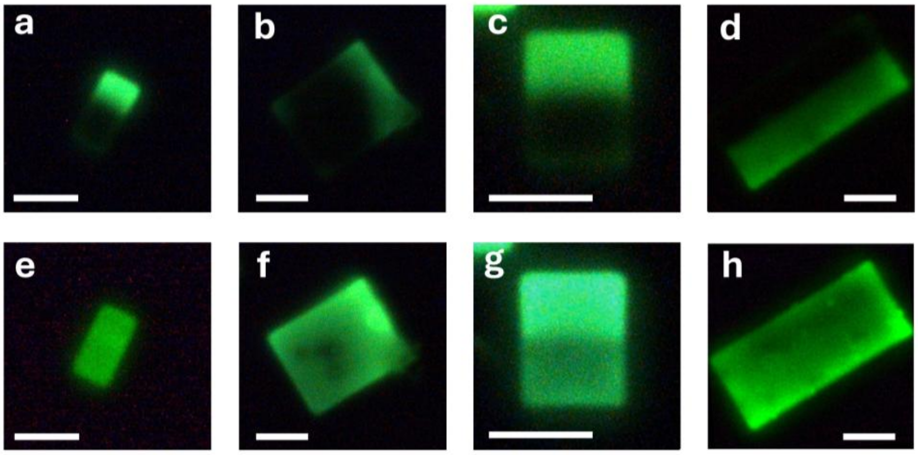

Figure S4: More examples of self-healing. (a), (b), (c), and (d) shows crystals right after FIB milling, and (e), (f), (g) and (h) shows the same particle after a healing period of a few days. All scale bars are 5 μm.

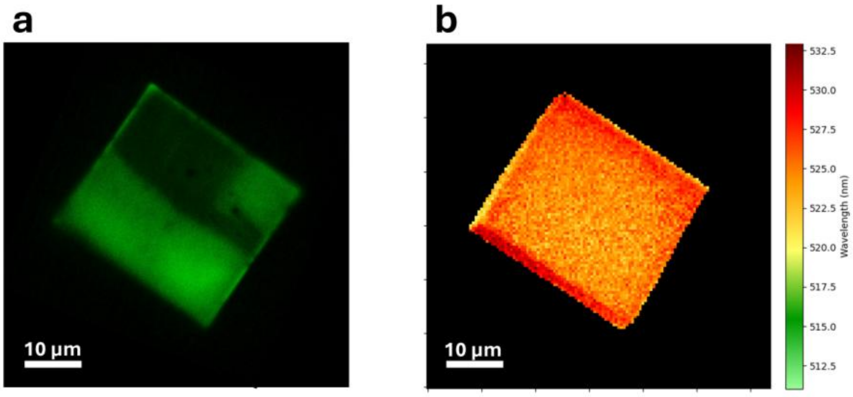

Figure S5: (a) PL of a large microcrystal after FIB milling showing reduced emission. (b) Cathodoluminescence peak wavelength of the same particle, showing no appreciable difference between the etched and the intact regions.

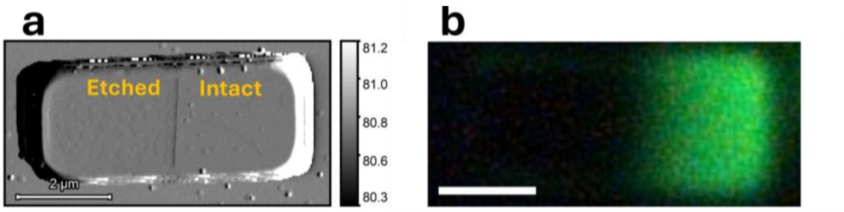

Figure S6: (a) AFM amplitude of the particle from Figure 3 along with its PL right after FIB milling in (b).

5. AFM Topography

The height distribution of the particles ranges from a few hundred nm up to a few microns. Figure s6 shows AFM measurements of a few representative particles.

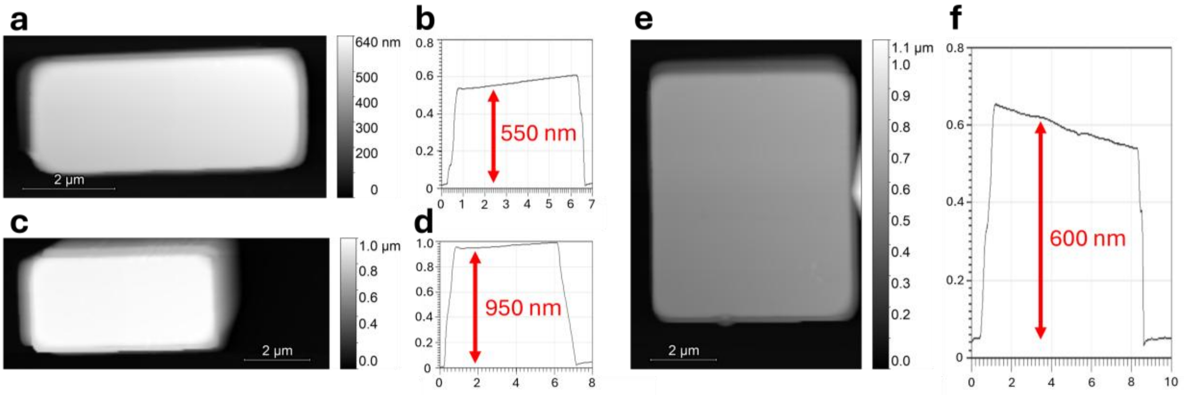

Figure S7: AFM topography of three particles and their corresponding line profiles. (a) The same particle from Figure 3 with a measured height of 550 nm shown in (b), (c-d) of shows a different particle with measured height of 950 nm, and (e-f) shows height of around 600 nm.

6. Surface reconstruction

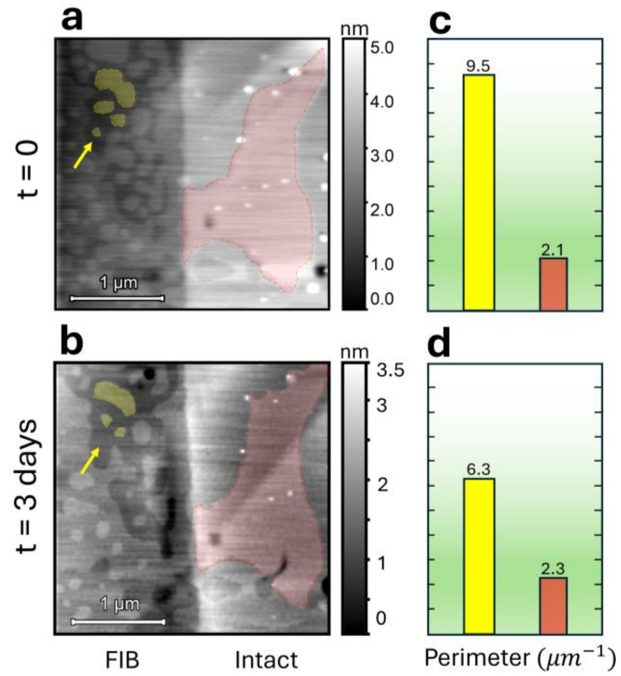

Figure S8: (a) AFM topography of a crystal's surface right after FIB milling (a) and after 3 days (b), showing a comparison between the two sides of the crystal. The overall terraces perimeter of the two sides is compared right after FIB (c) and after 3 days (d), showing a clear reduction for the damaged half, indicative of crystal rearrangement. The optical response of this particle is presented in Figure 4e-f in the main text.

7. Density Functional Theory Calculations

We performed first-principles density functional theory (DFT) calculations using the Vienna Ab initio Simulation Package (VASP).[3,4] The calculations were carried out within the generalized gradient approximation (GGA) employing the PBEsol exchange–correlation functional. Spin–orbit coupling (SOC) was included to account for relativistic effects. The Projector Augmented-Wave method (PAW) formalism was used[5] with a plane-wave cutoff energy of 520 eV and a Γ-centered k-point spacing of 0.25 $Å^{-1}$. In the direction perpendicular to the slab (z-direction), only a single k-point was sampled. Surface models were constructed as slab geometries separated by a vacuum region of 25 Å along the z-axis. We model the 100 and the 310 surface after the surface terminations present in the bottom of the two faceted wells, as visualized in Figure S8. The 100 surface contains 120 atoms, and the 310 slab model contains 100 atoms. Due to the asymmetry of the slab terminations, dipole corrections were applied in all surface calculations.

Structural relaxations were performed using the conjugate gradient algorithm as implemented in VASP. The lattice parameters were kept fixed, and the atomic positions in the bottom three layers of the slab were constrained to represent bulk-like behavior. Convergence is defined by the residual forces being below 0.05 eV/Å. Pre- and post-processing of the calculations were carried out using the Atomic Simulation Environment (ASE).[6]

We calculate the surface formation energy of the 100 and 310 slabs according to:

$$\bar{\gamma} = \frac{E_{slab}^{DFT} - N_{slab} \cdot E_{bulk}^{DFT}}{2A}$$

Where E_{slab}^{DFT} is the total DFT energy [eV] of the slab, N_{slab} is the number of atoms in the slab, E_{bulk}^{DFT} is the DFT energy per atom [eV/atom] of the bulk reference structure, and A is the surface area (in Å²), obtained from the in-plane lattice parameters a and b. The factor of two accounts for the two equivalent surfaces present on the top and the bottom in the slab model. The surface formation energies of the pristine slabs are presented in Figure S8.

For the defected structures, all defects are introduced within the top layer of the slab model, as can be seen in Figure S8. Defect formation energies in the surface slabs were computed using the following expressions:

$$\Delta E_{form}^{V_{Br}} = \left(E_{V_{Br}} + E_{Br}\right) - E_{pristine}$$
$$\Delta E_{form}^{Br_i} = E_{Br_i} - \left(E_{pristine} + E_{Br}\right)$$
$$\Delta E_{form}^{Pb_i} = E_{Pb_i} - \left(E_{pristine} + E_{Pb}\right)$$

Where $\Delta E_{form}^{V_{Br}}$ etc. are the formation energies of the defects, $E_{pristine}$ is the total DFT energy [eV] of the pristine slab structure, $E_{V_{Br}}$ etc. are the total DFT energies [eV] of the defected structures and E_{Br} and E_{Pb} are the reference energies of elemental Pb and Br [eV]. All defects are charge-neutral. All VASP input and output files associated with this work are publicly available through the DTU Data repository, ensuring full reproducibility and reusability of the results.[7]

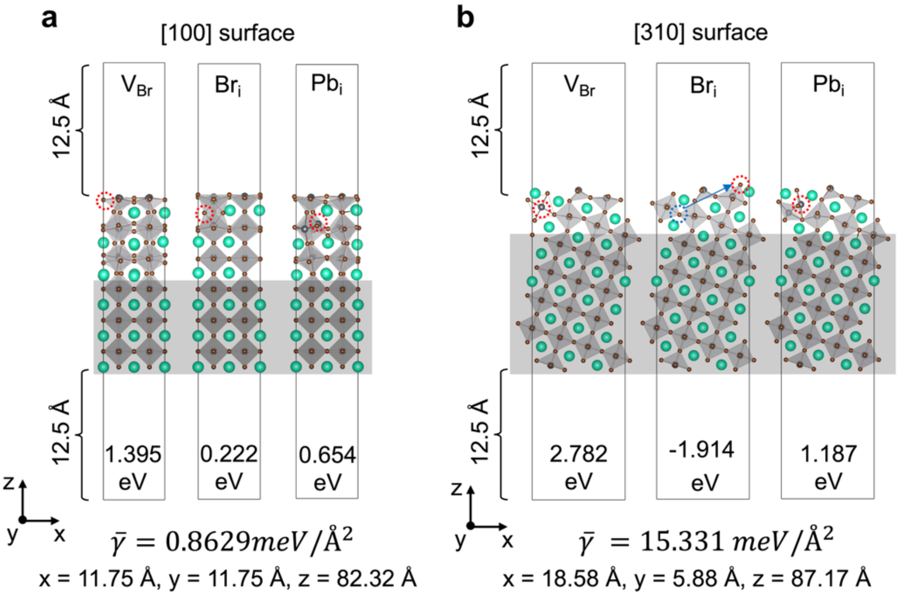

Figure S9: DFT calculations of the surface energies (γ) and defects formation energies of (100) in (a) and (310) in (b). The gray overlay represents the fixed layers on the bottom of the slab. Red dotted circles show the relaxed position of the defect in the slab, which is close to the initial positioning of the defect in the initial structure. In the case of the Bri defect in the 310 slab, the blue dotted circle shows the initial defect position. It can be seen that a large structural relaxation occurs, until the Br positions itself on the top of the slab, completing a stable position of a full octahedral.

References:

[1] Shorubalko, Ivan, Lex Pillatsch, and Ivo Utke. "Direct–write milling and deposition with noble gases." *Helium Ion Microscopy*. Cham: Springer International Publishing, 2016. 355-393.

[2] Eder, Katja, et al. "A multi-ion plasma FIB study: Determining ion implantation depths of Xe, N, O and Ar in tungsten via atom probe tomography." *Ultramicroscopy* 228 (2021): 113334.

[3] Kresse, Georg, and Jürgen Hafner. "Ab initio molecular dynamics for liquid metals." Physical review B 47, no. 1 (1993): 558.

[4] Kresse, Georg, and Jürgen Furthmüller. "Efficient iterative schemes for ab initio total-energy calculations using a plane-wave basis set." Physical review B 54, no. 16 (1996): 11169.

[5] Kresse, Georg, and Daniel Joubert. "From ultrasoft pseudopotentials to the projector augmented-wave method." Physical review b 59, no. 3 (1999): 1758.

[6] Hjorth Larsen, A., Jørgen Mortensen, J., Blomqvist, J., Castelli, I.E., Christensen, R., Dułak, M., Friis, J., Groves, M.N., Hammer, B., Hargus, C. and Hermes, E.D., 2017. The atomic simulation environment—a Python library for working with atoms. Journal of Physics: Condensed Matter, 29(27), p.273002.

[7] 10.11583/DTU.31371037
